# Supplementary material for: UV photochemistry of the L-cystine disulfide bridge in aqueous solution investigated by femtosecond X-ray absorption spectroscopy
Source: Nat Commun. 2024 Oct 13;15:8838. doi: 10.1038/s41467-024-52748-x (PMC11471820; doi:10.1038/s41467-024-52748-x)
Supplement: Supplementary file 1 — Supplementary Information [file 41467_2024_52748_MOESM1_ESM.pdf]

UV photochemistry of the L-cystine disulfide bridge in aqueous solution investigated by femtosecond X-ray absorption spectroscopy

Miguel Ochmann[a,†], Jessica Harich[a,†], Rory Ma[b], Antonia Freibert[a,*], Yujin Kim[c], Madhusudana Gopannagari[c], Da Hye Hong[c], Daewoong Nam[b,d], Sangsoo Kim[b], Minseok Kim[b], Intae Eom[b,d], Jae Hyuk Lee[b,d], Briony A. Yorke[e], Tae Kyu Kim[c,*] and Nils Huse[a,*]

[a]*Department of Physics, University of Hamburg and Center for Free-Electron Laser Science, 22761 Hamburg, Germany*

[b]*Pohang Accelerator Laboratory, POSTECH, Pohang 37673, Republic of Korea*

[c]*Department of Chemistry, Korea Advanced Institute of Science and Technology (KAIST), Daejeon 34141, Republic of Korea*

[d]*Photon Science Center, Pohang University of Science and Technology, Pohang 37673, Republic of Korea*

[e]*School of Chemistry, University of Leeds, Leeds, LS2 9JT, United Kingdom*

[†]*equal contribution*

Supplementary Information

- Suppl. Note 1: Lineshape analysis and potential reaction products
- Suppl. Fig. 1: Spectroscopic lineshapes and transitions of potential reaction products
- Suppl. Table 1: Lineshape parameters for the thiyl transition
- Suppl. Table 2: Lineshape parameters for the lowest sulfur-1s transitions of L-cystine

- Suppl. Note 2: Bond energies

- Suppl. Note 3: Composition of delay scans
- Suppl. Fig. 2: Composition of the delay scans at 2466.8 eV and 2468.2 eV

- Suppl. Note 4: Kinetic Model
- Suppl. Fig. 3: Schematic of the kinetic model
- Suppl. Table 3: Optimal fit parameters for the kinetic model

- Suppl. Note 5: Optimized structures and TDDFT vs. ADC(2)-x calculations
- Suppl. Fig. 4: Optimized structures.
- Suppl. Table 4: Comparison of transitions calculated with TDDFT and ADC(2)-x

- Suppl. Note 6: Estimates of two-photon excitation yields

Suppl. Note 1: Lineshape analysis and potential reaction products

The energetically lowest induced absorption at a time delay of 300 fs has Lorentzian lineshape that extends to about 2469 eV. This lineshape has a signal strength of 8.18 % of its peak value at 2468.2 eV where we have probed the emergence of a delayed induced absorption feature.

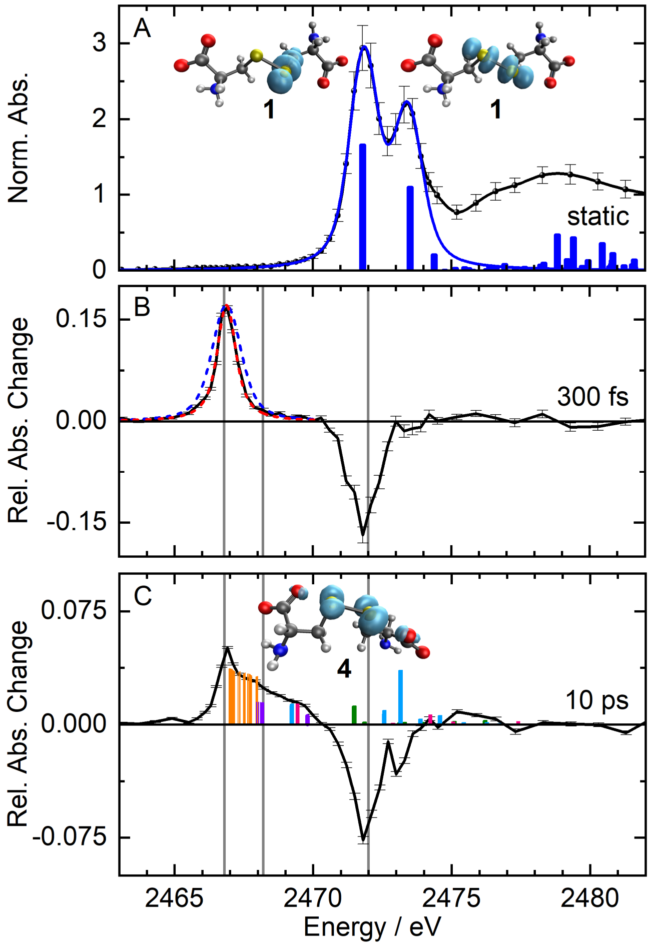

Supplementary Figure 1: Spectroscopic lineshapes and transitions of potential reaction products. (error bars represent standard deviations of the 600 measurement values acquired in 10 s from which each data point was calculated. **A** Static S K-edge X-ray absorption spectrum of L-cystine (**1**, black) with calculated lowest vertical excitation energies (blue sticks) and corresponding isosurface plots of the difference electron attachment densities (pale blue) and a fit of a Voigt profile to the lowest two transitions. **B** Differential spectrum 300 fs after 267-nm excitation (black) with a fit of a Voigt profile (dashed red curve, fixed Gaussian-FWHM of 0.35 eV) to the lineshape at 2466.8 eV. For comparison a Voigt profile (dashed blue curve) using the parameters obtained from the fit of a Voigt profile to the static spectrum in panel A is shown. **C** Differential spectrum 10 ps after 267-nm excitation (black) with calculated rotamer transitions of L-cysteinyl thiyl (orange) and L cysteinyl perthiyl (purple) radicals. The calculated transitions for triplet L-cystine (**4**, light blue, with corresponding isosurface plot of the difference electron attachment density) as well as for cationic (pink) and for anionic (green) L-cysteine are plotted as well. The vertical lines (grey) indicate the energies at which the delay scans in Suppl. Fig. 3 were recorded. Source data are provided as a Source Data file.

By fitting a Voigt-profile (red dashed curve in Suppl. Fig. 1B) to the lineshape at about 2466.8 eV with a Gaussian spectral bandwidth of the Si(111) double-crystal monochromator of $\mathrm{FWHM}_{\mathrm{G}}^{\mathrm{Si(111)}} = 0.35\ \mathrm{eV}$, we can extract the Lorentzian lifetime-broadened width of the lineshape at a value of $\mathrm{FWHM}_{\mathrm{L}} = 0.66\ \mathrm{eV}$. The complete set of optimal parameters are listed in Suppl. Table 1.

We have also considered additional photoproducts the structure of which can be optimized. Their lowest sulfur-1s transitions are plotted in Suppl. Fig. 1C. In light blue are the transitions of the calculated energetically lowest triplet state the energy of which is 4 eV above the singlet ground-state of L-cystine. It is energetically accessible upon photoexcitation with 4.6 eV (267 nm) photons but the differential spectrum at 300 fs precludes a triplet-state interpretation. The initial photoproduct is clearly the L-cysteine thiyl radical. A secondary triplet-state formation after the initial ultrafast sulfur-sulfur bond cleavage seems highly unlikely for energetic reasons because the S-S or C-S bond dissociation energies (BDE) are much lower ($BDE_{S\text{-}S}$ = 2.9 eV[1], $BDE_{C\text{-}S}$ = 2.3 eV[2]). Spectroscopically, the differential absorption spectra at 3 ps and 10 ps cannot be explained by triplet L-cystine as a secondary photoproduct because (i) the induced absorption below the lowest triplet sulfur-1s transition cannot be accounted for and (ii) the strongest calculated triplet transition at about 2473 eV would counterbalance the bleach signal observed at that position such that we would expect a positive absorbance change.

In pink and green we plotted the transitions for the L-cystine cation and anion, respectively that could form if the bond cleavage is not homolytic in nature. The sum of these transitions cannot explain an early photoproduct spectrum nor the differential absorption spectra at 3 p and 10 ps time delay. Photoionization of the parent compound at earliest time delays can also be ruled out because no corresponding absorption signal is observed at early delays.

Suppl. Table 1: Optimal Voigt parameters for the lineshape at 2466.8 eV with a fixed monochromator bandwidth of $\mathrm{FWHM}_\mathrm{G}^{\mathrm{Si(111)}} = 0.35$ eV, yielding a Lorentzian width of $\mathrm{FWHM_L} = 0.66$ eV.

Model	Voigt
Equation	y = nfl_voigt(x,y0,xc,A,wG,wL)
y0	0 ± 0
xc	2.46688 ± 8.32465E-6
$\mathrm{FWHM}_\mathrm{G}^{\mathrm{Si(111)}}$	3.5E-4
$\mathrm{FWHM_L}$	6.56986E-4 ± 2.454046E-5
A	2.05093E-4 ± 4.34588E-6
Reduced Chi-Sqr	3.67882
R-Square (COD)	0.99322
Adj. R-Square	0.99254

Suppl. Table 2: Voigt fit parameters for the first two transitions (Peak1 and Peak2) of the static absorption spectrum, with a fixed $\mathrm{FWHM_L} = 0.66$ eV resulting in a lineshape $\mathrm{FWHM}_\mathrm{G}^{\mathrm{sum}} = 0.86$ eV.

Model	Voigt	
Equation	y = nlf_voigt(x,y0,xc,A,wG,wL);	
Plot	Peak1	Peak2
y0*	0 ± 0	0 ± 0
xc	2.47183 ± 3.29714E-6	2.47344 ± 5.34421E-6
A	0.00444 ± 1.81103E-5	0.00322 ± 1.82845E-5
$\mathrm{FWHM}_\mathrm{G}^{\mathrm{sum}}$	8.61679E-4 ± 7.54195E-6	8.61679E-4 ± 7.54195E-6
$\mathrm{FWHM_L}$	6.56986E-4 ± 0	6.56986E-4 ± 0
Reduced Chi-	2.87053E-4	
R-Square (COD)	0.99968	
Adj. R-Square	0.99964	

The static absorption spectrum of L-cystine has a distinctly broader shape. A fit of a Voigt profile of the first two transitions with the natural linewidth fixed to $\mathrm{FWHM_L} = 0.66$ eV is shown in Suppl. Fig. 1A (blue). These absorption lineshapes feature Gaussian width of $\mathrm{FWHM}_\mathrm{G}^{\mathrm{sum}} = 0.86$ eV. The complete set of optimal parameters are listed in Suppl. Table 2. From this Gaussian broadening we can calculate the Gaussian broadening due to spectral inhomogeneity of the sample absorption according to

$$\mathrm{FWHM}_\mathrm{G}^{\mathrm{sample}} = \sqrt{\left(\mathrm{FWHM}_\mathrm{G}^{\mathrm{sum}}\right)^2 - \left(\mathrm{FWHM}_\mathrm{G}^{\mathrm{Si(111)}}\right)^2} = 0.79\ \mathrm{eV} \quad (1)$$

A Voigt profile with the lineshape parameters of the L-cystine sulfur-1s absorption as a solid red curve is plotted in Suppl. Fig. 1B to illustrate the substantial broadening in the parent compound’s sulfur-1s spectrum.

Suppl. Note 2: Bond energies

TDDFT calculations show that rotations around the C-S bond of the thiyl radical will shift the lowest sulfur-1s transition to higher energy compared to the optimized geometry as the distribution of orange sticks in Suppl. Fig. 1C illustrates. We have also considered the formation of a triplet state configuration when the lone-pair orbitals of the sulfur atoms align (light blue sticks in Suppl. Fig. 1B). However, not only is this geometry quite particular, its calculated energy is 4 eV above the singlet ground-state of L-cystine, significantly higher than the S-S or C-S bond dissociation energies (BDE_{S-S} = 2.9 eV[1], BDE_{C-S} = 2.3 eV[2]). A secondary triplet formation after the initial ultrafast sulfur-sulfur bond cleavage thus seems highly unlikely. Our data furthermore discourages secondary triplet formation since their strongest calculated transition would cancel out the bleach signal observed at that position (~ 2473 eV). Heterolytic splitting of the disulfide bond would yield L-cysteinyl cations and L-cysteinyl anions with sulfur-1s transitions shown as pink and green sticks, respectively. We consider the formation of a cation-anion pair as secondary product unlikely, as such products often have a higher energy barrier than the thiyl radicals that emerge from a homolytic bond cleavage as shown exemplary for dimethyl disulfide (DMDS) using the following enthalpies of formation and electron affinity:

$$\Delta_f H_g^{298^\circ}(\mathrm{DMDS}) = -5.8\ \frac{\mathrm{kcal}}{\mathrm{mol}}, \text{ from Hawari et al.}^3 \quad (2)$$

$$\Delta_f H_g^{298^\circ}(\mathrm{CH_3S}) = 29.8\ \frac{\mathrm{kcal}}{\mathrm{mol}}, \text{ from Nicovichet al.}^4 \quad (3)$$

$$\Delta_f H_g^{298^\circ}(\mathrm{CH_3S^+}) = 206\ \frac{\mathrm{kcal}}{\mathrm{mol}}, \text{ from Butler et al.}^5 \quad (4)$$

$$EA(\mathrm{CH_3S}) = 43\ \frac{\mathrm{kcal}}{\mathrm{mol}}, \text{ from Moran and Ellison}^6 \quad (5)$$

For the homolytic cleavage of the disulfide bond we obtain:

$$\mathrm{DH(S-S)} = 2 \cdot \Delta_\mathrm{f} H_\mathrm{g}^{298^\circ}(\mathrm{CH_3S}) - \Delta_\mathrm{f} H_\mathrm{g}^{298^\circ}(\mathrm{DMDS}) = 2 \cdot 29.8 - (-5.8)\frac{\mathrm{kcal}}{\mathrm{mol}} = 65.4\frac{\mathrm{kcal}}{\mathrm{mol}} \quad (6)$$

And for the heterolytic cleavage of the disulfide bond we obtain:

$$\mathrm{DH(S-S)} = \Delta_\mathrm{f} H_\mathrm{g}^{298^\circ}(\mathrm{CH_3S^+}) + (\Delta_\mathrm{f} H_\mathrm{g}^{298^\circ}(\mathrm{CH_3S}) - \mathrm{EA(CH_3S)}) - \Delta_\mathrm{f} H_\mathrm{g}^{298^\circ}(\mathrm{DMDS})$$

$$= 206 + 29.8 - 43 - (-5.8)\frac{\mathrm{kcal}}{\mathrm{mol}} = 225\frac{\mathrm{kcal}}{\mathrm{mol}} \quad (7)$$

The heterolysis of the disulfide bond thus requires a dissociation energy of 225 kcal/mol (9.7 eV), which is significantly above the used excitation energy of 4.6 eV (267 nm).

Suppl. Note 3: Composition of delay scans

The delay scans at the spectral positions 2466.8 eV and 2468.2 eV have been averaged from multiple scans (Suppl. Fig. 2A): The delay scan at 2466.8 eV is composed from two separate measurements, one covering the range from -0.5 ps to 10 ps, the second covering the time range until 800 ps. Both time traces were scaled to the appropriate spectral feature at 3 ps. The delay scan at 2468.2 eV is a weighted average of two and three separate measurements, one covering the range from -0.5 ps to 10 ps, two traces covering the time range until 800 ps. All time traces were scaled to the appropriate spectral feature at 3 ps.

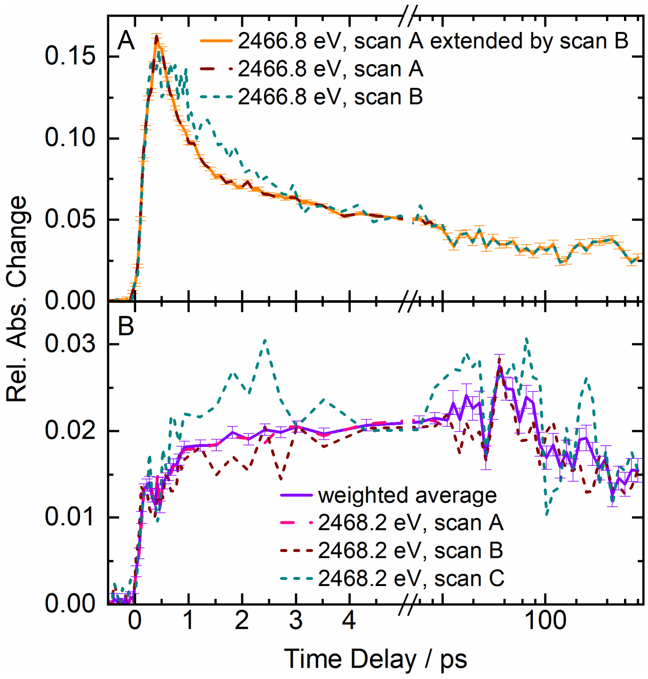

Suppl. Figure 2: Composition of the delay scans at the most prominent product transitions. Error bars represent standard deviations of the 600 measurement values acquired in 10 s from which each data point was calculated. **A**: The delay scan at 2466.8 eV (orange) is composed from two separate measurements: the first one ranging from-0.5 ps to 10 ps (dashed brown), the second one ranging up to 800 ps (dotted turquoise) **B** The delay trace at 2468.2 eV (purple) is a variance-weighted average of three separate measurements: From -0.5 up to 10 ps three scans (dashed pink, dotted dark brown and dotted turquoise) are averaged, from 10 ps to 800 ps two scans are averaged (dotted dark brown and dotted turquoise). Source data are provided as a Source Data file.

Suppl. Note 4: Kinetic Model

The experimental observations have been modelled by a rate-equations as shown in Suppl. Fig. 3: After excitation into the exited state (N_1), the exited state population decays resulting in the formation of thiyl radicals (N_2) by symmetrically breaking the disulfide bond. Except for a small part of the produced radicals that are stable on the observed time scales (rate constant $k_{23} = 0$), the population of the N_2 decays biexponentially (rate constants k_{21} and $k_{22} > k_{21}$). This decay re-populates the electronic ground state surface of L-cystine at high vibrational energy. Most of the population of this vibrationally excited state (VES, N_3) can dissipate the excess energy, leading to the recovery of the parent molecule (N_0) with rate constant k_{31}.The remaining population undergoes cleavage of the C-S bond (k_{32}), yielding L-cysteinyl perthiyl radicals and L-cysteinyl carbonyl radicals (N_4). Part of the perthiyl radical population is stable on the observed time scales (rate constant $k_{42} = 0$), the rest undergoes in-cage recombination to reform the parent disulfide (rate constant k_{41}).

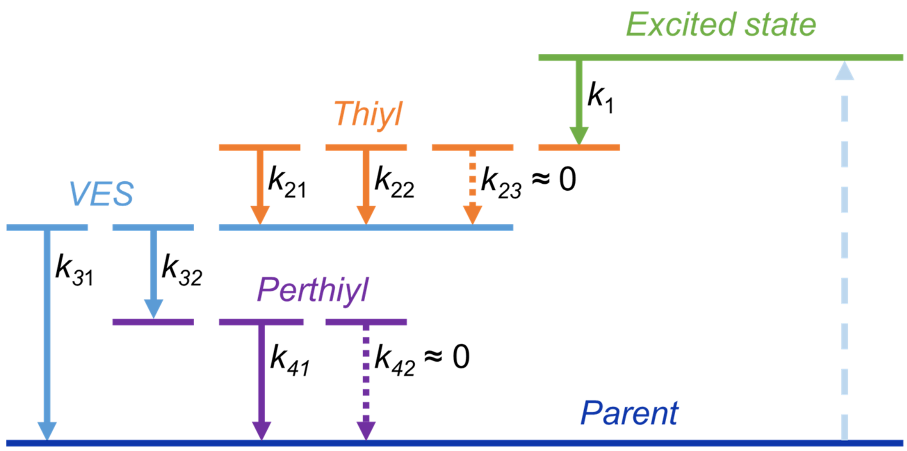

Suppl. Figure 3: Schematic of the developed kinetic fitting model. After the 267 nm excitation, the population of the Excited state (N_1, light green) decays with the rate constants k_1, into the N_2 state (Thiyl, orange). The N_2 state decays into a vibrationally highly excited state of the parent molecule N_3 (VES, light blue) on three rate constants k_{21}, k_{22} and $k_{23} = 0$. The N_3 state decays on two rate constants, the k_{31} decay reforms the ground state parent (N_0, blue), the k_{32} decay yields the perthiyl (N_4, purple). The N_4 decays with two rate constants k_{41}, reforming the parent molecule, and $k_{42} = 0$.

The populations N_1, N_2, N_3, N_4 and N_0 according to the above rate-model are provided in the following. We define $k_1 := t_1^{-1}$ and $k_{\mathrm{ij}} := t_{\mathrm{ij}}^{-1}$ to link time constants and rate constants. All exponential decay terms are convolved with a Gaussian instrument response function, yielding multiplication with an error function:

$$e_{\mathrm{ij}} := \exp\left(-k_{\mathrm{ij}}(t - t_0) + \frac{\sigma^2 k_{\mathrm{ij}}^2}{2}\right) \cdot \mathrm{err}(k_{\mathrm{ij}}), \qquad (8)$$

with the error function defined as

$$\mathrm{err}(k_{\mathrm{ij}}) := \frac{1}{2}\left(1 + \mathrm{erf}\left[\frac{1}{\sqrt{2}}\left(\frac{t-t_0}{\sigma} - \sigma k_{\mathrm{ij}}\right)\right]\right) \qquad (9)$$

In the particular case of $k_{ij} \to 0$ we define

$$\mathrm{Err} := \mathrm{err}(0) = \frac{1}{2}\left(1 + \mathrm{erf}\left[\frac{1}{\sqrt{2}}\left(\frac{t-t_0}{\sigma}\right)\right]\right) \qquad (10)$$

We then obtain the following solutions for the populations.

$\boldsymbol{N_1(t)}$:

The population $N_1(t)$ of the initially excited state is given by $N_1(t) = e_1$ with e_1 defined analogously to eq. 8 with k_1 replacing k_{ij}.

$\boldsymbol{N_2(t)}$:

The population $N_2(t)$ of the thiyl radical, which grows at a rate k_1 consists of three sub-ensembles N_{21}, N_{22}, N_{23} that decay with the respective rates k_{21}, k_{22} and k_{23}. The yields of these populations are q_{21}, q_{22} and q_{23} with $q_{23} = 1 - q_{21} - q_{22}$:

$$N_2(t) = \sum_{i=1}^{3} q_{2\mathrm{i}}\, N_{2\mathrm{i}}(t) \quad \text{where} \quad N_{2\mathrm{i}}(t) = k_1 \left[\frac{e_1}{(k_{2\mathrm{i}}-k_1)} + \frac{e_{2\mathrm{i}}}{(k_1-k_{2\mathrm{i}})}\right] \tag{11}$$

Since the population of N_2 does not fully decay in the observed time range we set $k_{23} \approx 0$.

$\boldsymbol{N_3(t)}$:

The population $N_3(t)$ of the intermediate vibrationally excited state grows with the rates k_{21} and k_{22} and consists of two sub-ensembles, N_{31} and N_{32}, that decay with rates k_{31} and k_{32}, respectively. The yields of these populations are q_{31} and q_{32} with $q_{32} = 1 - q_{31}$:

$$N_3(t) = \sum_{j=1}^{2} \sum_{i=1}^{3} q_{3\mathrm{j}}\, q_{2\mathrm{i}} N_{3\mathrm{j}}^{2\mathrm{i}}(t) \ \text{ where} \tag{12}$$

$$N_{3\mathrm{j}}^{2\mathrm{i}}(t) = k_1 k_{2\mathrm{i}} \left[\frac{e_1}{(k_{2\mathrm{i}}-k_1)(k_{3\mathrm{j}}-k_1)} + \frac{e_{2\mathrm{i}}}{(k_1-k_{2\mathrm{i}})(k_{3\mathrm{j}}-k_{2\mathrm{i}})} + \frac{e_{3\mathrm{j}}}{(k_1-k_{3\mathrm{j}})(k_{2\mathrm{i}}-k_{3\mathrm{j}})}\right] \tag{13}$$

Note that for $k_{23} \longrightarrow 0$ it follows that $N_{3\mathrm{j}}^{23}(t) \longrightarrow 0$.

$\boldsymbol{N_4(t)}$:

The population $N_4(t)$ of the perthiyl radical grows with the rate k_{32} and consists of two sub-ensembles, N_{41} and N_{42}, that decay with the respective rates k_{41} and k_{42}. The yields of these populations are q_{41} and q_{42} with $q_{42} = 1 - q_{41}$:

$$N_4(t) = \sum_{j=1}^{2} \sum_{i=1}^{3} q_{4j}\, q_{32} q_{2i} N_{4j}^{2i}(t) \ \text{ where} \tag{14}$$

$$N_{4\mathrm{j}}^{2\mathrm{i}}(t) = k_1 k_{2\mathrm{i}} k_{32} \left[\frac{e_1}{(k_{2\mathrm{i}} - k_1)(k_{32} - k_1)(k_{4\mathrm{j}} - k_1)} + \frac{e_{2i}}{(k_1 - k_{2\mathrm{i}})(k_{32} - k_{2\mathrm{i}})(k_{4\mathrm{j}} - k_{2\mathrm{i}})} + \frac{e_{32}}{(k_1-k_{32})(k_{2i}-k_{32})(k_{4j}-k_{32})} + \frac{e_{4j}}{(k_1-k_{4j})(k_{2i}-k_{4j})(k_{32}-k_{4j})}\right] \tag{15}$$

Similar to before it is $N_{4\mathrm{j}}^{23}(t) \longrightarrow 0$ for $k_{23} \longrightarrow 0$. Moreover, since the population of N_4 does not fully decay over the observed time range, we set $k_{42} \approx 0$.

$\boldsymbol{N_0(t)}$:

Finally, the population $N_0(t)$ of the ground state recovers with the rates k_{31} and k_{41}:

$$N_0(t) = \sum_{i=1}^{3} q_{31} q_{2\mathrm{i}}\, N_{03}^{2\mathrm{i}}(t) + \sum_{j=1}^{2} \sum_{i=1}^{3} q_{4\mathrm{j}}\, q_{32} q_{2\mathrm{i}} N_{04\mathrm{j}}^{2\mathrm{i}}(t) \tag{16}$$

Here, $N_{03}^{2\mathrm{i}}(t)$ is defined by

$$N_{03}^{2\mathrm{i}}(t) = k_1 k_{2\mathrm{i}} k_{31} \left(\frac{\mathrm{Err}}{k_1 k_{2\mathrm{i}} k_{31}} - \frac{e_1}{k_1(k_{2\mathrm{i}}-k_1)(k_{31}-k_1)} - \frac{e_{2\mathrm{i}}}{k_{2\mathrm{i}}(k_1-k_{2\mathrm{i}})(k_{31}-k_{2\mathrm{i}})} - \frac{e_{31}}{k_{31}(k_1-k_{31})(k_{2\mathrm{i}}-k_{31})}\right) \tag{17}$$

where, as before, $N_{03}^{23}(t) \longrightarrow 0$ for $k_{23} \longrightarrow 0$.

Further, $N_{04\mathrm{j}}^{2\mathrm{i}}(t)$ is definbed by

$$N_{04\mathrm{j}}^{2\mathrm{i}}(t) = k_1 k_{2\mathrm{i}} k_{32} k_{4\mathrm{j}} \left(\frac{\mathrm{Err}}{k_1 k_{2\mathrm{i}} k_{32} k_{4\mathrm{j}}} - \frac{e_1}{k_1(k_{2\mathrm{i}} - k_1)(k_{32} - k_1)(k_{4\mathrm{j}} - k_1)} - \frac{e_{2\mathrm{i}}}{k_{2\mathrm{i}}(k_1 - k_{2i})(k_{32} - k_{2\mathrm{i}})(k_{4\mathrm{j}} - k_{2\mathrm{i}})} - \frac{e_{32}}{k_{32}(k_1-k_{32})(k_{2\mathrm{i}}-k_{32})(k_{4\mathrm{j}}-k_{32})} - \frac{e_{4\mathrm{j}}}{k_{4\mathrm{j}}(k_1-k_{4\mathrm{j}})(k_{2\mathrm{i}}-k_{4\mathrm{j}})(k_{32}-k_{4\mathrm{j}})}\right) \tag{18}$$

where $N_{04\mathrm{j}}^{23}(t) \longrightarrow 0$ for $k_{23} \longrightarrow 0$ and $N_{042}^{23}(t) \longrightarrow 0$ for $k_{42} \longrightarrow 0$.

This set of equations was fit to the experimental delay scans in Fig. 3, with the signal evolution at 2466.8 eV, 2468.2 eV, and 2472.0 eV being proportional to N_2, N_4, and N_0, respectively.

We fit all parameters globally to every curve except for the zero delay points (t_0). A factor of two is applied to N_2 because splitting the disulfide bond of one L-cystine molecule yields two thiyl radicals. In the case of the delay scan at 2468.2 eV a spectral contribution of the Lorentzian lineshape at 2466.8 eV of 8.18 % N_2 needs to be included.

$$\Delta A(2466.8\ \mathrm{eV}) = 2A_{N2} \cdot N_2 \tag{19}$$

$$\Delta A(2468.2\ \mathrm{eV}) = A_{N4} \cdot N_4 + 0.0818 \cdot 2A_{N2} \cdot N_2 \tag{20}$$

$$\Delta A(2472.0\ \mathrm{eV}) = A_{N0} \cdot N_0 \tag{21}$$

Suppl. Table 3: Optimized parameters of the rate-equation model. Parameters marked with a star are globally fit to the three delay scans.

Parameter	2466.8 eV	2468.2 eV	2472.0 eV
**t0 / ps**	-0.02277 ± 0.00786	-0.00553 ± 0.01479	-0.01786 ± 0.02213
**s* / ps**	0.08734 ± 0.00589	0.08734 ± 0.00589	0.08734 ± 0.00589
**t1* / ps**	0.14457 ± 0.02304	0.14457 ± 0.02304	0.14457 ± 0.02304
**t21* / ps**	0.40859 ± 0.04604	0.40859 ± 0.04604	0.40859 ± 0.04604
**t22* / ps**	5.65655 ± 0.51402	5.65655 ± 0.51402	5.65655 ± 0.51402
**t31* / ps**	0.63336 ± 0.26165	0.63336 ± 0.26165	0.63336 ± 0.26165
**t32* / ps**	0.45137 ± 0.06406	0.45137 ± 0.06406	0.45137 ± 0.06406
**t41* / ps**	268.245 ± 164.501	268.245 ± 164.501	268.245 ± 164.501
**q21***	0.66872 ± 0.01846	0.66872 ± 0.01846	0.66872 ± 0.01846
**q22***	0.19253 ± 0.01084	0.19253 ± 0.01084	0.19253 ± 0.01084
**q31***	0.65997 ± 0.04258	0.65997 ± 0.04258	0.65997 ± 0.04258
**q41***	0.42262 ± 0.11288	0.42262 ± 0.11288	0.42262 ± 0.11288
**A_thiyl (N2)***	0.1203 ± 0.0081	0.1203 ± 0.0081	0.1203 ± 0.0081
**A_perthiyl (N4)***	0.06546 ± 0.00867	0.06546 ± 0.00867	0.06546 ± 0.00867
**A_bleach (N0)***	-0.13716 ± 0.00774	-0.13716 ± 0.00774	-0.13716 ± 0.00774
**f_thiyl**	1 ± 0	0.0818 ± 0	0 ± 0
**f_perthiyl**	0 ± 0	1 ± 0	0 ± 0
**f_bleach**	0 ± 0	0 ± 0	1 ± 0
**Reduced Chi-Sqr***		3.83933	
**R-Square (COD)**	0.99464	0.96935	0.91457
**R-Square (COD)***		0.99532	
**Adj. R-Square***		0.99503	

Suppl. Note 5: Optimized structures and TDDFT vs. ADC(2)-x calculations

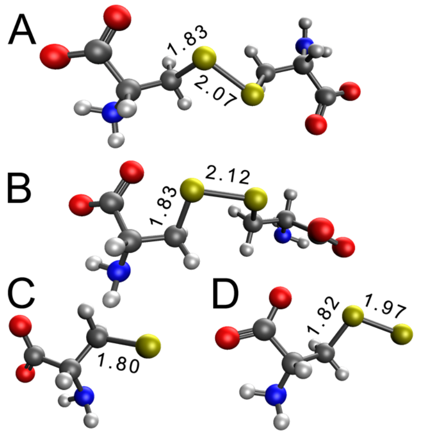

Suppl. Fig 4: Optimized ground-state structures.
A L-cystine, **B** triplet L-cystine, **C** L-cysteinyl-thiyl radical and **D** L-cysteinylperthiyl radical. All C-S and S-S bond lengths are given in Ångström.

The optimized molecular geometries of the parent disulfide, the triplet L-cystine as well as the thiyl and perthiyl radicals are shown in Figure S4 with the S-C and S-S bond lengths annotated in Ångström. The triplet geometry shows a lengthening of the S-S bond length compared to the parent molecule, as well as a different dihedral angle.

We have confirmed the validity of using TD-DFT to calculate the transitions at the sulfur K-edge by another method: Equilibrium structure optimizations of all molecular species were performed at the second-order Møller-Plesset perturbation theory[7] (MP2) using the Dunning correlation consistent basis set[8] aug-cc-pvtz, where vibrational frequency calculations confirmed the finding of true energetic minima. All ground state calculations were carried out using the quantum chemistry software package Gaussian.[9]

The X-ray absorption transitions were simulated using the extended variant of the algebraic-diagrammatic construction scheme of second order[10] (ADC(2)-x) with the aug-cc-pvdz basis set exploiting the core-valence separation (CVS) approximation as implemented in the quantum chemistry program package Q-Chem 5.4.[11] The transition energies of all calculated species were shifted by -9.83 eV in order to match the experimental spectra. The comparison is shown in Suppl. Table 4, we note that the results obtained with TDDFT and ADC(2)-x are, apart from a different global shift, of good general agreement, confirming the TDDFT calculations as sufficient. This is an important conclusion for potential future calculations concerning bigger molecules.

Suppl. Table 4: Comparison of calculated sulfur 1s transitions using TDDFT (shifted by 52.09 eV) or ADC(2)-x (shifted by -9.83 eV).

	**TDDFT transition energy / eV (shift = 52.09 eV)**	**ADC(2)-x transition energy / eV (shift = -9.83 eV)**
**Parent**	2471.8027	2471.8054
		2471.8055
	2473.5209	2473.2222
		2473.2222
**Thiyl**	2466.9987	2466.491
	2473.3993	2474.2969
	2473.6953	2474.4419
**Perthiyl**	2468.0901	2468.0067
	2469.8162	2469.9324
**Triplet**	2469.2345	2469.3364
	2469.2368	2469.3365
	2472.5691	2472.7469
	2472.5696	2472.7469

Suppl. Note 6: Estimates of two-photon excitation yields

Two-photon absorption (TPA) of the pump pulse ($E_{\lambda=267\mathrm{nm}} \approx$ 4.6 eV, τ = 100 fs) can lead to the excitation of otherwise transparent solvents. For the case of liquid water, 2PA is already observed above 6 eV despite the fact that a water molecule in gas-phase cannot be excited at this photon energy. An upper bound of TPA by liquid water can be calculated using the incident pump fluence of $\phi = 1 \cdot 10^{17}\ \mathrm{ph/cm^2}$ equivalent to $75\ \mathrm{mJ/cm^2}$. The sample absorption and the TPA cross-section of water[12], $\sigma_2 \sim 10^{-50}\ \mathrm{cm^4 s}$, to predict that yield of two-photon excited water molecules: $\sigma_2 \phi^2/\tau = 0.1\ \%$. With a two-photon excitation energy of 9.2 eV, an equal probability for ionization (i.e. solvated electrons) and homolytic OH cleavage exists in liquid water.[13] Within 55.56 M water we can thus expect 28 mM of homolytic cleavage and ionization products, respectively. Using Avogadro's constant, the number of molecules per volume can be calculated from the concentration. For 28 mM of a chemical species $2.8 \cdot 6.02\ \cdot\ 10^{-3}$ particles/nm³ exist (either molecules or electrons). This density corresponds to $59\ \mathrm{nm}^3$ per particle of which the third root yields the average distance between a particular species, i.e. $d_{\mathrm{avg}}(28\ \mathrm{mM}) = 3.9\ \mathrm{nm}$. In the same way, the distance between two species can eb calculated.

We can calculate the concentration of thiyl radicals at 300 fs from Suppl. Fig. 1 by considering our spectral normalization scheme: We have normalized all absorption measurements to the edge-jump of the sulfur absorption. This absorption step is proportional to the number of sulfur atoms which absorb X-ray photons. We can assume that the energetically lowest bleach signal (2472.90 eV) at 300 fs is not significantly perturbed by photoproduct absorption. We can therefore approximate the excitation yield by dividing the change in absorption by the total sample absorption. This means that we have excited 0.15 / 3 = 5 % excitation yield. Given the disulfide molecules of 100 mM, we are creating 5 mM of thiyl radical pairs or 10 mM of thiyl radicals. Of this thiyl concentration 20 % or 2 mM still exist after 20 ps (c.f. Fig. 3 in the main article).

At 20 ps in one cubic nanometer an estimated combined number of thiyl radicals and a reactive species of $(2.8 + 0.2) \cdot 6.02 \cdot 10^{-3}$ particles is present, also yielding an average distance between a thiyl and a reactive species of $d = 3.9\ \mathrm{nm}$. Using the diffusion coefficients of the hydrated electron $D_{\mathrm{e-}}$ and the hydroxyl radical $D_{\mathrm{OH\cdot}}$, we can calculate the diffusion time until those reactive species statistically encounter a thiyl radical (which is considerably larger which is why we neglect thiyl diffusion here):

$$D_{\mathrm{e-}} = 4.9 \cdot 10^{-9}\,\frac{\mathrm{m}^2}{\mathrm{s}}, \text{ from Schmidt et al. [14]} \tag{22}$$

$$D_{\mathrm{OH\cdot}} = 2.31 \cdot 10^{-9}\,\frac{\mathrm{m}^2}{\mathrm{s}}, \text{ from Ashton et al. [15]} \tag{23}$$

The mean squared distance in three dimensions can be described as $d^2 = 6Dt$.[16] Thus we obtain diffusion time constants $t = \frac{d^2}{6D}$ for the hydroxyl anion and the hydroxyl radical of

$$t_{\mathrm{e-}} = \frac{(3.9 \cdot 10^{-9})^2\ \mathrm{m}^2}{6 \cdot 4.9 \cdot 10^{-9}\ \mathrm{m}^2}\ \mathrm{s} = 0.52\ \mathrm{ns} \tag{24}$$

$$t_{\mathrm{OH\cdot}} = \frac{(3.9 \cdot 10^{-9})^2\ \mathrm{m}^2}{6 \cdot 2.31 \cdot 10^{-9} \mathrm{m}^2}\ \mathrm{s} = 1.1\ \mathrm{ns} \tag{25}$$

These numbers can be taken as upper bounds because the photoproducts due to TPA of the solvent will also geminately and diffusively recombine, thereby further increasing the distance between reactive species. We do not observe significant change in the thiyl signals which suggests that even time delays of 0.52 ns and beyond, the reaction between solvated electrons and photoproducts is

insignificant. Moreover, our findings of the L-cystine photochemistry are based differential absorption spectra recorded at time delays of 10 ps or shorter and time scans that are vary very little after 0.1 ns. An encounter between two chemical species does not mean that a chemical reaction actually occurs. We can consider reactions involving solvated electrons by considering bimolecular reaction rates of the hydrated electrons with thiyl radicals to form radical anions. Second-order rate constants will depend on the specific molecule and for solvated electrons on pH among other factors. A typical rate constant for reactions between small sulfur-containing molecules and solvated electrons have been reported[17] at a values of $k_{\mathrm{e}^-} = 5 \cdot 10^9\ \mathrm{M^{-1}s^{-1}}$. Similar values can also be found for reactions with OH radicals.[18] We can calculated the concentration c of products due to the reaction of photoproducts, e.g. thiyl radicals) with hydrated electrons at a delay time t:

$$\begin{aligned} c(t = 500\ \mathrm{ps}) &= t \cdot k_{\mathrm{e}^-} \cdot [\mathrm{e}^-][\mathrm{Thiyl}] \\ &= 5 \cdot 10^{-10}\ \mathrm{s}\ \cdot\ 5 \cdot 10^9\ \mathrm{M^{-1}s^{-1}} \cdot\ 0.028\ \mathrm{M}\ \cdot\ 0.002\ \mathrm{M} \\ &= 0.14\ \mathrm{mM} \end{aligned} \tag{26}$$

This concentration is 10-fold smaller than the smallest photoproduct concentrations we recorded. This result is also an upper bound because of the assumption that the concentration of hydrated electrons stays constant over the time delay of 500 ps after generation at $t = 0$. We are thus ruling out significant reaction yields between photoproducts and solvated electrons/hydroxyl radicals.

Supplementary References

1. Roux, M. V. *et al.* Experimental and Computational Thermochemical Study of Sulfur-Containing Amino Acids: l-Cysteine, l-Cystine, and l-Cysteine-Derived Radicals. S−S, S−H, and C−S Bond Dissociation Enthalpies. *J. Phys. Chem. B* **114**, 10530–10540 (2010).
2. Benson, S. W. Thermochemistry and kinetics of sulfur-containing molecules and radicals. *Chem. Rev.* **78**, 23–35 (1978).
3. Hawari, J. A., Griller, D. & Lossing, F. P. Thermochemistry of perthiyl radicals. *J. Am. Chem. Soc.* **108**, 3273–3275 (1986).
4. Nicovich, J. M., Kreutter, K. D., Van Dijk, C. A. & Wine, P. H. Temperature-dependent kinetics studies of the reactions bromine atom (2P3/2)+ hydrogen sulfide. tautm. mercapto+ hydrogen bromide and bromine atom (2P3/2)+ methanethiol. tautm. methylthiol+ hydrogen bromide. Heats of formation of mercapto and methylthio radicals. *J. Phys. Chem.* **96**, 2518–2528 (1992).
5. Butler, J. J., Baer, T. & Evans, S. A. Jr. Energetics and structures of organosulfur ions: CH3SSCH3+, CH3SS+, C2H5S+, and CH2SH+. *J. Am. Chem. Soc.* **105**, 3451–3455 (1983).
6. Moran, S. & Ellison, G. B. Photoelectron spectroscopy of sulfur ions. *J. Phys. Chem.* **92**, 1794–1803 (1988).
7. Møller, Chr. & Plesset, M. S. Note on an Approximation Treatment for Many-Electron Systems. *Phys. Rev.* **46**, 618–622 (1934).
8. Dunning, T. H. Gaussian basis sets for use in correlated molecular calculations. I. The atoms boron through neon and hydrogen. *J. Chem. Phys.* **90**, 1007–1023 (1989).
9. Frisch, M. J. *et al.* Gaussian 16, Revision C.01. (2016).
10. Trofimov, A. B. & Schirmer, J. An efficient polarization propagator approach to valence electron excitation spectra. *J. Phys. B At. Mol. Opt. Phys.* **28**, 2299–2324 (1995).

11. Shao, Y. *et al.* Advances in molecular quantum chemistry contained in the Q-Chem 4 program package. *Mol. Phys.* **113**, 184–215 (2015).

12. Dragonmir, A., McInerney, J. G. & Nikogosyan, D. N. Femtosecond measurements of two-photon absorption coefficients at λ= 264 nm in glasses, crystals, and liquids. *Appl. Opt.* **41**, 4365–4376 (2002).

13. Elles, C. G., Shkrob, I. A., Crowell, R. A. & Bradforth, S. E. Excited state dynamics of liquid water: Insight from the dissociation reaction following two-photon excitation. *J. Chem. Phys.* **126**, 164503 (2007).

14. Schmidt, K. H., Han, P. & Bartels, D. M. Temperature dependence of solvated electron diffusion in water and water-d2. *J. Phys. Chem.* **96**, 199–206 (1992).

15. Ashton, L., V. Buxton, G. & R. Stuart, C. Temperature dependence of the rate of reaction of OH with some aromatic compounds in aqueous solution. Evidence for the formation of a π-complex intermediate? *J. Chem. Soc. Faraday Trans.* **91**, 1631–1633 (1995).

16. Atkins, P. W., Paula, J. D. & Keeler, J. *Atkins' Physical Chemistry*. (Oxford University Press, 2023).

17. Hoffman, M. Z. & Hayon, E. One-electron reduction of the disulfide linkage in aqueous solution. Formation, protonation, and decay kinetics of the RSSR-radical. *J. Am. Chem. Soc.* **94**, 7950–7957 (1972).

18. Bonifacic, M., Schaefer, K., Moeckel, H. & Asmus, K. D. Primary steps in the reactions of organic disulfides with hydroxyl radicals in aqueous solution. *J. Phys. Chem.* **79**, 1496–1502 (1975).
