# Supplementary material for: UV photochemistry of the L-cystine disulfide bridge in aqueous solution investigated by femtosecond X-ray absorption spectroscopy
Source: Nat Commun. 2024 Oct 13;15:8838. doi: 10.1038/s41467-024-52748-x (PMC11471820; doi:10.1038/s41467-024-52748-x)
Supplement: Supplementary file 3 — Description of Additional Supplementary Files [file 41467_2024_52748_MOESM3_ESM.pdf]

Description of Additional Supplementary Files:

Supplementary Data 1: Atomic coordinates of optimized molecular structures
